# *BAK1* gene variation and abdominal aortic aneurysms—results may have been prematurely overrated

**DOI:** 10.1002/humu.21324

**Published:** 2010-09-28

**Authors:** Sébastien Küry, Fabrice Airaud, Philippe Piloquet, Stéphane Bézieau

**Affiliations:** CHU de Nantes, pôle de Biologie, service de Génétique MédicaleNantes, cedex 1, France

We have read with much interest the article Gottlieb et al. [2009] about *BAK1* (MIM♯ 600516), as well as the epistolary exchange with Dr. Hatchwell that followed [Gottlieb et al., 2010; Hatchwell, 2010]. Our reading was all the more careful that the message delivered by the authors was strong, and was largely echoed and amplified in many scientific and nonscientific journals. Much noise was made about this article presented as a revolution in genetics, hanging a question mark over the dogma of a unique genome shared by all body cells. We were therefore eager to know all the details about this outstanding finding.

In the first place, we thought that it was quite an original idea to simultaneously compare the genotypes of the *BAK1* gene between abdominal aortic tissue, matched nondiseased tissue, and blood leukocytes. Yet, we were quickly annoyed by many inaccuracies and unclear explanations that seemed to distort the authors' message.

The most confusing point fits in the commentary on the article by Hatchwell [2010]. We did not understand why the authors had not compared the same type of nucleic acid between blood (DNA) and matching aortic tissues (RNA). And yet, in their reply to Dr. Hatchwell, the authors state that “blood and tissue *BAK1* genomic sequences were identical” [Gottlieb et al., 2010]. It is astounding that the authors forgot to mention this crucial observation in their original article, because it runs counter to the theory of a genome varying according to the tissue, that is, the very message widely spread by the media. Indeed, this observation means that the two different genotypes reported by Gottlieb et al. in Table 2 of their article do not correspond to differences between tissues but rather to differences between types of nucleic acid (DNA vs. RNA). It therefore excludes the authors' postulate of a mosaicism and of an allelic selection due to some environmental conditions.

The alternative hypothetic mechanism of somatic mutations seems also to be discarded. Indeed, Gottlieb et al.'s Table 2 suggests that allelic variants at position 42 (rs1051911), 52 (rs1051912), and 81 (rs1051913) are likely in very strong or even complete linkage disequilibrium, because only two haplotype combinations are represented (major haplotype CA**G**/GC**T**/AT**C** vs. variant haplotype CA**A**/GC**C**/AT**T**). By the way, it is not clear whether the genotypes reported in Gottlieb et al.'s Table 2 were found in a homozygous state; it seems odd that the sequences of the aortic tissues do not exhibit traces of the major allelic variants, which are largely predominant in blood (of note, rs1051912 and rs1051913 variant alleles were never found in any of the HapMap or SNP500 cancer populations). Assuming that the genotypes are really homozygous anyway, the results in Table 2 of Gottlieb et al.'s article mean that all the aortic DNA samples tested would have to derive from a primary DNA that would have systematically undergone the same triple modification. To our knowledge, even in cancer, where recurrent somatic mutations in hot spots are well known (e.g., mutation V600E in *BRAF* or mutations in codons 12 and 13 of *KRAS* in colorectal cancer) [Oliveira et al., 2007], a triple nucleotidic modification that would systematically affect three distinct and nonadjacent codons has never been described.

The explanation of the difference between DNA and RNA sequences observed by the authors must therefore lie at the RNA level. Yet, it cannot be explained by RNA editing. The work of Gottlieb et al. [2009] indeed highlights changes G>A (codon 42), T>C (codon 52), and C>T (codon 81), whereas in the mechanism of RNA editing described to date, ADARs and cytidine deaminases induce A>G and C>T change, respectively [Mattick and Mehler, 2008]. Thus, only the variant observed in codon 81, that is, the one corresponding to a synonymous change, could be affected by RNA editing, unless the authors point to a new mechanism.

**Figure 1:**
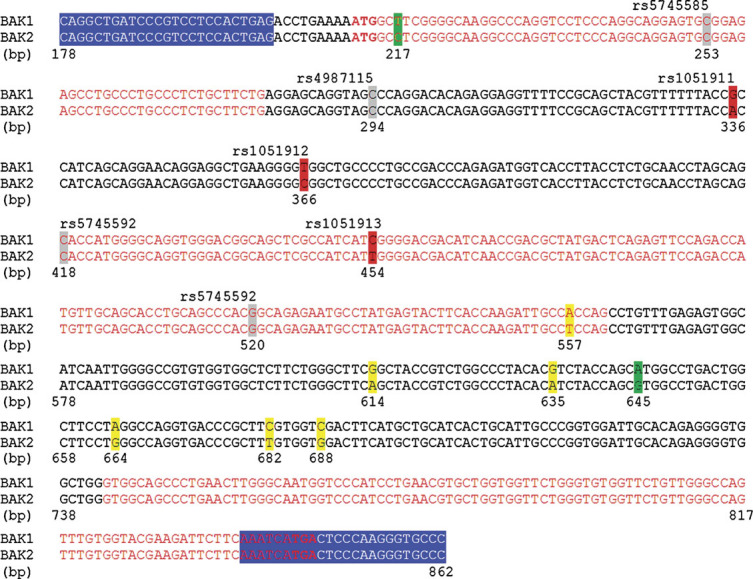
Comparison of *BAK1* cDNA (NCBI reference: NM_001188.3) and *BAK2* genomic sequences (NCBI reference: NG_000850). The primers used by Gottlieb et al. [2009] for the RT-PCR of *BAK1* are highlighted in blue. Sequences are numbered in base pairs using the same nomenclature as Gottlieb et al. [2009]. The three nucleotides highlighted in red are the three SNPs found variant by the authors in their original article, whereas the four remaining SNPs that they cited are highlighted in gray. The nucleotides highlighted in green are the two differences between *BAK1* and *BAK2* cited by Gottlieb et al. [2010] in their reply to Hatchwell [2010] in order to justify the absence of amplification of *BAK2*. The other differences between *BAK1* and *BAK2*, which were not mentioned by Gottlieb et al., are highlighted in yellow.

**Figure 2:**
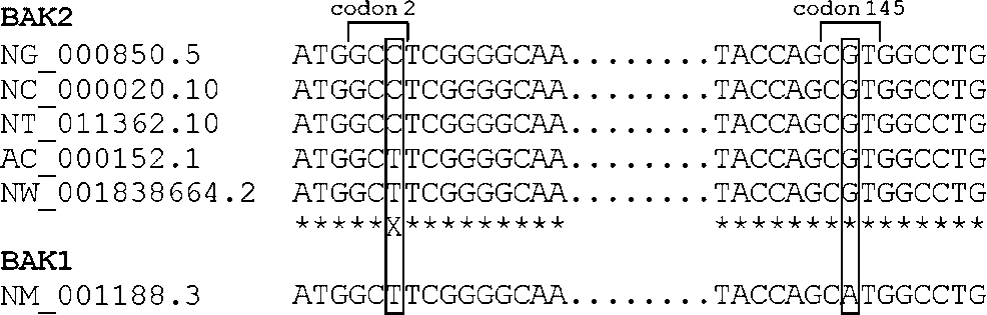
Comparison of the different sequences retrieved from NCBI for *BAK2* gene. Focus on the two variants in codons 2 and 145 used by Gottlieb et al. [2010] to refute a crosscontamination between *BAK1* and *BAK2* sequences. We show here that two of the five *BAK2* reference sequences presented do not differ from *BAK1* at codon 2.

A more plausible hypothesis remains the crosscontamination between *BAK1* transcript and genomic DNA of pseudogene *BAK2* (*BAK1P1*) suggested by Hatchwell [2010]. Gottlieb et al. [2010] excluded that *BAK1* cDNA sequences of abdominal aortic tissues may have been contaminated by *BAK2* sequences, because of the RNAse treatment used for reverse transcription. They consider thereby that *BAK2* exists in its genomic form only [Gottlieb et al., 2010]. However, according to the databank reference sequence XM_002348050, *BAK2* would have been predicted according to similarities to 1 mRNA, 18 ESTs, and 5 proteins. The fact that one supporting evidence is an mRNA suggests that an mRNA transcript and even a translation is not unlikely for *BAK2*. This would confirm the hypothesis of a sequencing artifact [Hatchwell, 2010], given that the RNA sequences of *BAK1* and *BAK2* differ exactly at the points of variation (single nucleotide polymorphisms [SNPs]) observed by Gottlieb et al. [2009] (Fig. 1). Besides, in order to refute the hypothesis of a contamination by a sequence other than *BAK1*, the authors use two variants concerning codons 2 (ATG/GC**C**/TCG for *BAK2* vs. ATG/GC**T**/TCG for *BAK1*) and 145 of both *BAK1* and *BAK2* that would enable to discriminate the two genes [Gottlieb et al., 2010]. The problem is that the variant at codon 2 is not present in all the *BAK2* sequences of reference found in the NCBI databank (Fig. 2), which makes very doubtful the validity of the reference sequence for *BAK2* and thus the demonstration of Gottlieb et al. [2009,2010]. Moreover, the authors do not comment on the six other variants that differ between the sequences of *BAK1* (XM_002348050) and *BAK2* (NG_000850), which would be useful to make a discrimination between the two gene sequences.

In conclusion, like Dr. Hatchwell, we are not convinced that the cDNA sequence presented by Gottlieb et al. [2009,2010] in the aortic tissues corresponds to *BAK1* and not to pseudogene *BAK2*. Our doubts are all the more strong because the five controls tested by the authors all carry the three rare variants rs1051911 (codon 42), rs1051912 (codon 52), and rs105193 (codon 81). Whenever these rare variants were truly predisposing to aneurysm, the probability to find them in all five controls would be very low, unless there had been a significant bias in the recruitment of the controls. Yet, even if the contamination was confirmed there would still remain one enigma: why would the authors find a homozygous variant sequence, because the oligonucleotidic primers that they used for their experiments perfectly match both *BAK1* and *BAK2* mRNA sequences? One explanation could be an allele-specific amplification due to an undescribed polymorphism in one of the primer sequences.

All in all, Gottlieb et al. [2009] contains an accumulation of inaccuracies and paradoxical results that put a doubt on their conclusions. We do not pretend that their interpretations are wrong, but we believe that there is too huge a contrast between the strong message delivered by the authors and the quality of the experiments and report that they proposed, which did not meet the high-level standard expected for a major work—considering also the obvious translation errors of original Table 2 of Gottlieb et al.'s article corrected in the Erratum published online on 22 November 2009. Consequently, we hope that the authors will extend their study in order to clarify the sequence variations.

It is also noteworthy that the quite neutral title of the article, “*BAK1* gene variation and abdominal aortic aneurysms,” does not reflect the revolutionary idea relayed by the media. This suggests that the authors probably did not expect such an impact for their work, and that their results might have been prematurely overrated.
